# Risks of ischaemic heart disease and stroke in meat eaters, fish eaters, and vegetarians over 18 years of follow-up: results from the prospective EPIC-Oxford study

**DOI:** 10.1136/bmj.l4897

**Published:** 2019-09-04

**Authors:** Tammy Y N Tong, Paul N Appleby, Kathryn E Bradbury, Aurora Perez-Cornago, Ruth C Travis, Robert Clarke, Timothy J Key

**Affiliations:** 1 Cancer Epidemiology Unit, Nuffield Department of Population Health, University of Oxford, Richard Doll Building, Oxford OX3 7LF, UK; 2 Clinical Trial Service Unit and Epidemiological Studies Unit, Nuffield Department of Population Health, Big Data Institute, University of Oxford, Oxford, UK

## Abstract

**Objective:**

To examine the associations of vegetarianism with risks of ischaemic heart disease and stroke.

**Design:**

Prospective cohort study.

**Setting:**

The EPIC-Oxford study, a cohort in the United Kingdom with a large proportion of non-meat eaters, recruited across the country between 1993 and 2001.

**Participants:**

48 188 participants with no history of ischaemic heart disease, stroke, or angina (or cardiovascular disease) were classified into three distinct diet groups: meat eaters (participants who consumed meat, regardless of whether they consumed fish, dairy, or eggs; n=24 428), fish eaters (consumed fish but no meat; n=7506), and vegetarians including vegans (n=16 254), based on dietary information collected at baseline, and subsequently around 2010 (n=28 364).

**Main outcome measures:**

Incident cases of ischaemic heart disease and stroke (including ischaemic and haemorrhagic types) identified through record linkage until 2016.

**Results:**

Over 18.1 years of follow-up, 2820 cases of ischaemic heart disease and 1072 cases of total stroke (519 ischaemic stroke and 300 haemorrhagic stroke) were recorded. After adjusting for sociodemographic and lifestyle confounders, fish eaters and vegetarians had 13% (hazard ratio 0.87, 95% confidence interval 0.77 to 0.99) and 22% (0.78, 0.70 to 0.87) lower rates of ischaemic heart disease than meat eaters, respectively (P<0.001 for heterogeneity). This difference was equivalent to 10 fewer cases of ischaemic heart disease (95% confidence interval 6.7 to 13.1 fewer) in vegetarians than in meat eaters per 1000 population over 10 years. The associations for ischaemic heart disease were partly attenuated after adjustment for self reported high blood cholesterol, high blood pressure, diabetes, and body mass index (hazard ratio 0.90, 95% confidence interval 0.81 to 1.00 in vegetarians with all adjustments). By contrast, vegetarians had 20% higher rates of total stroke (hazard ratio 1.20, 95% confidence interval 1.02 to 1.40) than meat eaters, equivalent to three more cases of total stroke (95% confidence interval 0.8 to 5.4 more) per 1000 population over 10 years, mostly due to a higher rate of haemorrhagic stroke. The associations for stroke did not attenuate after further adjustment of disease risk factors.

**Conclusions:**

In this prospective cohort in the UK, fish eaters and vegetarians had lower rates of ischaemic heart disease than meat eaters, although vegetarians had higher rates of haemorrhagic and total stroke.

## Introduction

Vegetarian and vegan diets have become increasingly popular in recent years, partly due to perceived health benefits, as well as concerns about the environment and animal welfare.^1^ In the United Kingdom, both the representative National Diet and Nutrition Survey 2008-12 and a 2016 Ipsos MORI survey estimated about 1.7 million vegetarians and vegans living in the country.^2^ ^3^ Evidence suggests that vegetarians might have different disease risks compared with non-vegetarians,^4^ but data from large scale prospective studies are limited, because few studies have recruited sufficient numbers of vegetarian participants.

For ischaemic heart disease, some but not all previous studies reported significantly lower risks of mortality from ischaemic heart disease in vegetarians than in non-vegetarians.^5^ ^6^ ^7^ In terms of incidence, the only previous study (the European Prospective Investigation into Cancer (EPIC)-Oxford) reported that vegetarians had a lower risk of ischaemic heart disease than non-vegetarians,^8^ but at the time of publication the study had an insufficient duration of follow-up to separately examine the risks in other diet groups (fish eaters and vegans).

For stroke, two previous reports,^5^ ^6^ including one that included EPIC-Oxford data,^6^ found no significant differences in risk of total stroke deaths between vegetarians and non-vegetarians. However, no previous studies have examined the incidence of stroke in relation to vegetarian diets, or have examined the main stroke types.

We report here the risks of both incident ischaemic heart disease and stroke in people with distinct dietary habits—that is, meat eaters, fish eaters and vegetarians (including vegans)—with a separate evaluation of ischaemic and haemorrhagic strokes, over 18 years of follow-up in the EPIC-Oxford study.

## Methods

### Study population and design

EPIC-Oxford is a prospective cohort study of about 65 000 men and women who were recruited across the UK between 1993 and 2001. Details of the recruitment process have been described previously.^9^ Individuals were recruited from either general practices or by postal questionnaire. The general practice recruitment method recruited 7421 men and women aged 35 to 59 who were registered with participating general practices, all of whom completed a full questionnaire on their diet, lifestyle, health characteristics, and medical history. The postal recruitment preferentially targeted vegetarians, vegans, and other people interested in diet and health, and recruited 57 990 participants aged 20 or older. A full questionnaire was mailed to all members of the Vegetarian Society and all surviving participants of the Oxford Vegetarian Study,^10^ and respondents were invited to provide names and addresses of relatives and friends who were also interested in receiving a questionnaire. A short questionnaire was also distributed to all members of the Vegan Society, enclosed in vegetarian and health food magazines, and displayed in health food shops; and a full questionnaire was subsequently mailed to all those who returned the short questionnaire. 

Despite the targeted recruitment of the postal method, about 80% of meat eaters in the cohort were recruited by post. Subsequently, a follow-up questionnaire was sent to participants in 2010, which asked similar questions on their diet and lifestyle, and participants returned the questionnaires between 2010 and 2013. A participant flowchart of the recruitment process and inclusion into this study is shown as supplementary figure 1. The study protocol was approved by a multicentre research ethics committee (Scotland A Research Ethics Committee) and all participants provided written informed consent.

### Assessment of diet group and diet

The full baseline questionnaire collected responses to four questions about consumption of meat, fish, dairy products, and eggs, in the form of “Do you eat any meat (including bacon, ham, poultry, game, meat pies, sausages)?” or similar for the other three food groups. These four questions were used to classify participants into meat eaters (participants who reported eating meat, regardless of whether they ate fish, dairy, or eggs), fish eaters (participants who did not eat meat but did eat fish), vegetarians (participants who did not eat meat or fish, but did eat one or both of dairy products and eggs), and vegans (participants who did not eat meat, fish, dairy products, or eggs). The follow-up questionnaire sent in 2010 included identical questions on consumption of meat, fish, dairy products, and eggs (yes/no). Therefore, at both baseline and follow-up, participants were classified into one of four diet groups: meat eaters, fish eaters, vegetarians, and vegans. Owing to the small number of vegans, vegetarians and vegans were combined as one diet group in the main analyses, but the two groups were examined separately for each outcome in secondary analyses.

The baseline questionnaire also included a semiquantitative food frequency section containing 130 items, which asked about dietary intake over the past year, and which was previously validated using 16 days (in four sets of four days) of weighed dietary records and selected recovery and concentration biomarkers.^11^ ^12^ ^13^ For calculation of food and nutrient intakes, the frequency of consumption of each food or beverage was multiplied by a standard portion size (mostly based on data from the UK Ministry of Agriculture, Fisheries, and Food)^14^ and nutrient content of each food or beverage (based on McCance and Widdowson’s food composition tables).^15^ Because our prespecified analysis plan was to examine disease risks associated with distinct dietary groups, the associations of individual foods and nutrients with risks were not assessed in this study, but information on intakes of foods and nutrients were used in descriptive and secondary analyses.

### Assessment of other characteristics

In addition to diet, the baseline questionnaire also asked questions on sociodemographic characteristics, lifestyle, and medical history, including questions on education level, smoking, physical activity, use of dietary supplements, and use of oral contraceptives or hormone replacement therapy in women. Socioeconomic status was categorised by use of the Townsend deprivation index,^16^ based on the participants’ postcodes. For physical activity, based on their responses to questions asked about their occupation and their time spent participating in activities including walking, cycling, and other physical exercises, participants were categorised by a validated physical activity index with four levels.^17^ Alcohol consumption was determined from responses to five items on the food frequency questionnaire. Questions relating to smoking and alcohol consumption were also asked on the follow-up questionnaire in 2010.

For biological measurements, body mass index was calculated from participants’ self reported height and weight at recruitment, which was previously found to be accurate compared with measured height and weight in a validation study of about 4800 participants.^18^ All participants were also asked at recruitment whether they were willing to have their blood pressure measured at their general practice and to provide a blood sample. Details of the procedures for blood pressure measurement and blood sample collection, which were conducted in subsets of the cohort, have been previously reported.^8^ ^19^ ^20^

### Outcome ascertainment

Participants were followed up via record linkage to records from the UK’s health service up to 31 March 2016. Outcomes of interest were ischaemic heart disease (codes 410-414 from ICD-9 (international classification of diseases, 9th revision) or codes I20-I25 from ICD-10), including acute myocardial infarction (ICD-9 410 or ICD-10 I21); and total stroke (ICD-9 430-431, 433-434, 436; or ICD-10 I60-I61, I63-I64), including ischaemic stroke (ICD-9 433-434 or ICD-10 I63) and haemorrhagic stroke (ICD-9 430-431 or ICD-10 I60-I61). Details of events, using the relevant ICD-9 or ICD-10 codes, were obtained from hospital records or death certificates.

### Exclusion criteria

Participants who were not resident in England, Wales, or Scotland (n=945) were excluded, as were those with no Hospital Episode Statistics data or NHS number (n=20). We also excluded participants who completed the short questionnaire only (n=7619); were younger than 20 (n=1) or older than 90 at recruitment (n=58); had no follow-up (were censored at or before the date of recruitment (eg, if they were living abroad), n=364); could not be traced by the NHS (n=14); had an unknown diet group (if they did not answer the relevant questions to be classified, n=132); had unreliable nutrient data (≥20% of food frequencies missing, or daily energy intakes <500 kcal or >3500 kcal for women or <800 kcal or >4000 kcal for men (1 kcal=4.18 kJ=0.00418 MJ), n=1219); had a self reported history of acute myocardial infarction, stroke, or angina at recruitment (n=6837); or had a date of diagnosis that preceded or equalled the date of recruitment (n=14).

### Statistical analyses

Baseline characteristics and food and nutrient intakes of the EPIC-Oxford participants were summarised by diet group. For self reported body mass index, and measures of blood pressure (systolic and diastolic blood pressure) and blood lipids (total cholesterol, high density lipoprotein cholesterol (HDL-C), non-HDL-C), the means and 95% confidence intervals are presented, after adjustment for sex and age at entry (in 5-year age groups), alcohol consumption (<1, 1-7, 8-15, ≥16 g/day), and physical activity (inactive, low activity, moderately active, very active, unknown).^17^

Cox proportional hazards regression models were used to estimate the hazard ratios and 95% confidence intervals for the associations between diet group (meat eaters, fish eaters, vegetarians including vegans) and each outcome of interest, with meat eaters as the reference group. For participants who completed both the baseline and follow-up questionnaire, diet group and relevant time varying covariates (smoking and alcohol consumption) were updated at follow-up. The underlying time variable was the age at recruitment to the age at diagnosis, death, or administrative censoring, whichever occurred first. For acute myocardial infarction or ischaemic heart disease, events were censored on the respective outcomes of interest. For total stroke, ischaemic stroke, and haemorrhagic stroke, events were censored on any stroke. All analyses were stratified by sex, method of recruitment (general practice or postal), and region (seven regions across the UK), and adjusted for year of recruitment (per year from 1994 to 1999), education (no qualifications, basic secondary (eg, O level), higher secondary (eg, A level), degree, unknown), Townsend deprivation index (quarters, unknown),^16^ smoking (never, former, light, heavy, unknown), alcohol consumption (<1, 1-7, 8-15, ≥16 g/day), physical activity (inactive, low activity, moderately active, very active, unknown), dietary supplement use (no, yes, unknown), and oral contraceptive use (no, yes ever, unknown) and hormone replacement therapy use (no, yes ever, unknown) in women. 

We used Wald tests to test for heterogeneity of risk between diet groups. The proportional hazards assumption was assessed on the basis of Schoenfeld residuals, and was not violated for the variables of interest in the adjusted model for either ischaemic heart disease or stroke (P>0.05 for all categories). Self reported history of high blood pressure (no, yes, unknown), high blood cholesterol (no, yes, unknown), diabetes (no, yes, unknown), and body mass index (<20, 20-22.5, 22.5-25, 25-27.5, ≥27.5, unknown) were assessed as potential physiological mediators, since these factors were known to be associated with vegetarian diets,^19^ ^20^ ^21^ ^22^ ^23^ as well as being established cardiovascular risk factors.^24^ Total fruit and vegetable intake, total dietary fibre, and total energy intake (each continuous) were assessed as possible relevant dietary factors. We assessed the effects of potential physiological mediators and possible relevant dietary factors by adding each variable one at a time to the previous model. An additional model was also fitted including all potential physiological mediators.

To estimate the population impact of vegetarian diets on cardiovascular health, we assessed the absolute risk difference for each outcome between meat eaters, fish eaters, and vegetarians. Predicted incidence and absolute risk differences were presented as per 1000 population over 10 years, and were estimated by use of hazard ratios and 95% confidence intervals expressed as floating absolute risks,^25^ ^26^ which do not alter the value of the hazard ratios but assign an appropriate 95% confidence interval to all groups, including the reference group (thereby allowing an estimation of the uncertainty in the effect size in the reference group). In meat eaters, predicted incidence over this time period of each outcome was calculated as (1−S_r_)×1000, where S_r_=(1−observed incidence in meat eaters)^10^, and represents the predicted 10 year survival (that is, non-incidence) in meat eaters. By subtracting this estimate of survival from 1, and multiplying by 1000, the resulting estimate represents incidence per 1000 population over 10 years. For all other diet groups, predicted incidence was calculated as (1−S_r_^HR^)×1000, where HR represents the hazard ratio or confidence intervals for each outcome in that diet group. By applying HR or confidence interval estimates in each diet group as an exponential to survival in the reference group, S_r_^HR^ represents the predicted 10 year survival rate in the each of the other diet groups. Absolute risk differences were then calculated as the crude differences between the predicted incidence per 1000 population over 10 years between each diet group and the meat eaters.

Additional sensitivity analyses included analyses using baseline diet group only, excluding participants with less than five years of follow-up, including participants recruited via the postal method only, censoring at age 70 or setting entry time at age 70 to evaluate possible differences by age at event, and performing multiple imputation (with 10 imputations) for missing covariates. The percentages of missing values in the covariates were 12.7% for the Townsend deprivation index, 10.9% for physical activity, 6.3% for education level, and less than 2% for each of the other covariates. We assessed heterogeneity in the associations between diet group and risk of ischaemic heart disease or stroke by sex, age at recruitment (<60 or ≥60 years), smoking status (never, former, or current), body mass index (<25 or ≥25), presence of risk factors (one or more of self reported history of high blood pressure, high blood cholesterol, or diabetes), and any long term treatment for any illness or condition (no, yes) by adding appropriate interaction terms to the Cox models and testing for statistical significance of interaction across strata using likelihood ratio tests. All analyses were performed with Stata version 14.1 or 15.1 (Stata Corp, TX, United States) and P values less than 0.05 were considered significant.

### Patient and public involvement

No members of the community or patients were involved in setting the research question or the outcome measures, nor were they involved in developing plans for recruitment, design, or implementation of the study. They were not asked to advise on interpretation or writing up of results. We are appreciative of our participants who, although not partners, were engaged in the progress of EPIC-Oxford through follow-up questionnaires. The results are disseminated to study participants through newsletters and the study website (www.epic-oxford.org/).

## Results

### Cohort characteristics

The analyses included 48 188 participants, of whom 28 364 had reported on diet both at baseline and at follow-up on average 14 years later. Of these participants with repeated measures of diet, 13 972 (96%) of 14 540 meat eaters at baseline remained meat eaters at follow-up. Of fish eaters and vegetarians at baseline, 2608 (57%) of 4555 and 6746 (73%) of 9269 were in the same diet group at follow-up, respectively.

Cohort characteristics of the study participants for each of the three diet groups (meat eaters, fish eaters, vegetarians including vegans) at baseline are presented in table 1 (and separately for vegetarians and vegans in supplementary table 1). Overall, non-meat eaters were younger and had a lower area level socioeconomic status than meat eaters, but were more highly educated, less likely to smoke, reported slightly lower alcohol consumption, were more physically active, and were more likely to report dietary supplement use. They were also less likely to report previous high blood pressure, high blood cholesterol, or diabetes; or receive long term treatment for illnesses. In women, non-meat eaters were more likely to report oral contraceptive use but less likely to report use of hormone replacement therapy. Non-meat eaters reported having lower body mass index and had lower measured blood pressure than meat eaters. For blood lipids, vegetarians had about 0.5 mmol/L lower plasma concentrations of total cholesterol and non-HDL-C than meat eaters. Fish eaters had slightly higher plasma concentrations of HDL-C than meat eaters, while vegetarians had slightly lower concentrations.

**Table 1:** Baseline characteristics of participants in different diet groups in the EPIC-Oxford study(n=48 188)

Characteristics	Diet group*		
	Meat eaters (n=24 428)	Fish eaters (n=7506)	Vegetarians (n=16 254)
**Sociodemographic characteristics**			
Age, years (mean (standard deviation))	49.0 (13.1)	42.1 (12.8)	39.4 (13.1)
Sex, women (number (%))	18 481 (75.7)	6186 (82.4)	12 232 (75.3)
Top socioeconomic quarter (number (%))†	5959 (28.0)	1431 (21.9)	3018 (21.2)
Higher education (number (%))	7374 (32.8)	3308 (46.2)	6698 (43.3)
**Lifestyle**			
Current smokers (number (%))	2955 (12.1)	764 (10.2)	1685 (10.4)
Alcohol consumption, g/day (mean (standard deviation))	10.1 (12.9)	10.0 (12.3)	9.3 (12.8)
Moderate/ high physical activity (number (%))	6752 (31.2)	2684 (40.2)	5849 (40.0)
Dietary supplement use (number (%))‡	13 295 (55.6)	4702 (64.1)	8961 (56.1)
**Medical history (number (%))**			
Prior high blood pressure	2938 (12.1)	549 (7.3)	935 (5.8)
Prior high blood cholesterol	1616 (6.6)	255 (3.4)	345 (2.1)
Prior diabetes	353 (1.4)	61 (0.8)	93 (0.6)
Receiving long term treatment for any illness	7022 (29.1)	1622 (21.9)	3077 (19.1)
Oral contraceptive use§	13 263 (72.2)	4959 (80.5)	9620 (79.0)
Hormone replacement therapy use§	4484 (24.6)	728 (11.9)	954 (7.9)
**Biological measurements (adjusted mean (95% CI))¶**			
Body mass index	24.1 (24.0 to 24.1)	23.1 (23.0 to 23.2)	23.0 (23.0 to 23.1)
Systolic blood pressure (mm Hg)	125.7 (125.4 to 126.1)	123.4 (122.7 to 124.2)	123.7 (123.2 to 124.2)
Diastolic blood pressure (mm Hg)	77.1 (76.9 to 77.3)	75.5 (75.0 to 76.0)	75.9 (75.6 to 76.2)
Total cholesterol (mmol/L)	5.50 (5.46 to 5.54)	5.31 (5.23 to 5.39)	4.98 (4.92 to 5.03)
HDL cholesterol (mmol/L)	1.32 (1.31 to 1.33)	1.35 (1.32 to 1.38)	1.29 (1.27 to 1.31)
Non-HDL cholesterol (mmol/L)	4.18 (4.14 to 4.22)	3.96 (3.88 to 4.04)	3.68 (3.62 to 3.74)

For high density lipoprotein (HDL) cholesterol, P value for heterogeneity was 0.002 between diet groups, and less than 0.001 for all other variables.

* Meat eaters were participants who reported eating meat, regardless of whether they ate fish, dairy, or eggs; fish eaters were participants who did not eat meat but did eat fish; vegetarians included vegans.

† Based on Townsend index.

‡ Defined as regularly taking any vitamins, minerals, fish oils, fibre, or other food supplements during the past 12 months.

§ In women only.

¶ Body mass index was based on self reported measures in the whole cohort. Blood pressure was measured in 8862 meat eaters, 1742 fish eaters, and 4364 vegetarians and vegans. Blood lipids were measured in 1985 meat eaters, 566 fish eaters, and 1109 vegetarians and vegans. Estimates were adjusted for the cross stratification of sex and age at entry (5-year age groups), alcohol consumption (<1, 1-7, 8-15, ≥16 g/day), and physical activity (inactive, low activity, moderately active, very active, unknown).

Food and nutrient intakes in the different diet groups are presented in table 2 and supplementary table 2 (separately for vegetarians and vegans). Intakes of most foods and nutrients differed between the dietary groups: for example, vegetarians had higher intakes of fruit and vegetables, legumes and soya foods, nuts, and dietary fibre, and had a lower intake of saturated fat (10% *v* 12% energy) and sodium than meat eaters.

**Table 2:** Food and nutrient intakes of participants in different diet groups in the EPIC-Oxford study (n=48 188)

Foods or nutrients	Diet group*		
	Meat eaters (n=24 428)	Fish eaters (n=7506)	Vegetarians* (n=16 254)
Foods			
Total meat and meat products (g/day)	76.2 (48.5)	—	—
Red and processed meat (g/day)	50.3 (38.9)	—	—
Poultry (g/day)	26.0 (21.7)	—	—
Total fish (g/day)	41.9 (29.1)	38.5 (33.3)	—
Dairy milk (mL/day)	324.2 (184.7)	274.0 (189.8)	232.3 (207.3)
Soya milk (mL/day)	5.9 (43.2)	21.1 (79.4)	54.5 (127.9)
Dairy cheese (g/day)	20.7 (18.6)	27.5 (24.2)	26.8 (25.4)
Total fresh fruit (g/day)	264.8 (207.5)	291.1 (228.0)	283.8 (239.2)
Total vegetables (g/day)	252.3 (131.8)	287.3 (148.7)	294.4 (163.2)
Legumes and soya foods (g/day)	28.9 (30.3)	58.1 (44.4)	74.4 (58.1)
Nuts and nut butter (g/day)	4.8 (9.3)	8.0 (12.1)	10.6 (16.2)
Nutrients			
Carbohydrates (% energy)	48.0 (6.2)	51.0 (6.5)	52.8 (6.8)
Protein (% energy)	16.9 (3.0)	14.6 (2.3)	13.6 (2.1)
Total fat (% energy)	31.6 (5.9)	30.8 (6.3)	30.2 (6.6)
Saturated fat (% energy)	11.5 (3.3)	10.6 (3.3)	10.2 (3.5)
Monounsaturated fat (% energy)	10.7 (2.3)	9.9 (2.4)	9.7 (2.6)
Polyunsaturated fat (% energy)	6.3 (1.9)	7.0 (2.2)	7.1 (2.5)
Dietary fibre (g/day)	18.8 (6.7)	21.2 (7.4)	22.1 (8.0)
Sodium (mg/day)	2773 (864)	2684 (864)	2664 (885)
Total energy (kJ/day)	8298 (2250)	7939 (2199)	7813 (2234)

Data are mean (standard deviation). For all variables, P values for heterogeneity was less than 0.001 between diet groups.

* Meat eaters were participants who reported eating meat, regardless of whether they ate fish, dairy, or eggs; fish eaters were participants who did not eat meat but did eat fish; and vegetarians included vegans.

### Association of diet groups with cardiovascular diseases

**Fig 1:**
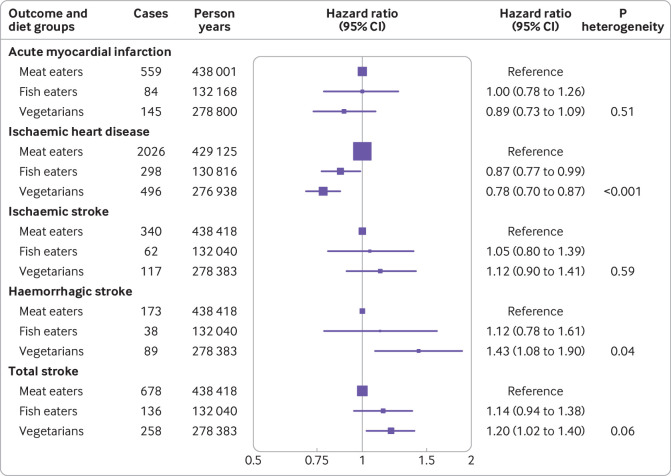
Rates of ischaemic heart disease and stroke in fish eaters and vegetarians (including vegans) compared with meat eaters in the EPIC-Oxford study (n=48 188). Meat eaters were participants who reported eating meat, regardless of whether they ate fish, dairy, or eggs; fish eaters were participants who did not eat meat but did eat fish; and vegetarians included vegans. Meat eaters were used as the reference group throughout. All analyses included age as the underlying time variable; were stratified by sex, method of recruitment (general practice or postal), and region (seven categories); and were adjusted for year of recruitment (per year), education (no qualifications, basic secondary (eg, O level), higher secondary (eg, A level), degree, unknown), Townsend deprivation index (quarters, unknown), smoking (never, former, light, heavy, unknown), alcohol consumption (<1, 1-7, 8-15, ≥16 g/day), physical activity (inactive, low activity, moderately active, very active, unknown), dietary supplement use (no, yes, unknown), and oral contraceptive and hormone replacement therapy use in women. P heterogeneity=significance of heterogeneity in risk between diet groups based on Wald tests. Box sizes are proportional to the number of cases in each group

Over 18.1 years of follow-up in 48 188 participants, 2820 cases of ischaemic heart disease (including 788 cases of acute myocardial infarction) and 1072 cases of total stroke (including 519 cases of ischaemic stroke and 300 cases of haemorrhagic stroke) were reported. Fish eaters (hazard ratio 0.87, 95% confidence interval 0.77 to 0.99) and vegetarians (0.78, 0.70 to 0.87) had lower rates of ischaemic heart disease than meat eaters (P_heterogeneity_<0.001 between all diet groups; fig 1). Conversely, vegetarians, but not fish eaters, had significantly higher rates of haemorrhagic stroke than meat eaters (1.43, 1.08 to 1.90; P_heterogeneity_=0.04) and higher rates of total stroke (1.20, 1.02 to 1.40; P_heterogeneity_=0.06). We saw no significant differences between diet groups for the risk of acute myocardial infarction or ischaemic stroke. When we assessed vegetarians and vegans separately, the point estimates for vegans were lower for ischaemic heart disease (0.82, 0.64 to 1.05) and higher for total stroke (1.35, 0.95 to 1.92) than meat eaters, but neither estimate was statistically significant, possibly because of the small number of cases in vegans, as indicated by the wide confidence intervals (supplementary table 3). 

When the roles of potential mediators of the associations were assessed, the associations between diet group and ischaemic heart disease were marginally attenuated when adjusted for self reported history of high blood pressure, high blood cholesterol, and diabetes, and for self reported body mass index. The lower rate of ischaemic heart disease in vegetarians was of borderline significance when adjusted for all potential mediators simultaneously (0.90, 0.81 to 1.00; supplementary table 4). For stroke, adjustments for potential mediators caused small changes in the hazard ratios; the association became slightly stronger for vegetarians after adjustment for self reported history of high blood pressure, and to a lesser extent when adjusted for history of diabetes or for body mass index. Additional adjustment for fruit and vegetable intake, total fibre, or total energy had little effect on the results.

### Absolute risk difference for vascular disease outcomes by diet group

Table 3 shows the absolute risks of ischaemic heart disease and total stroke in the three diet groups. By comparing the absolute risk difference of each outcome by diet group, for an individual of the cohort’s average age (44.7 years) and other characteristics (supplementary table 1), and not accounting for competing risk from other diseases or influences from other external factors, vegetarian diets were associated with 10 fewer (95% confidence interval 6.7 to 13.1 fewer) cases of ischaemic heart disease per 1000 population over 10 years than meat eaters. Conversely, vegetarian diets were associated with three more (95% confidence interval 0.8 to 5.4) cases of total stroke.

**Table 3:** Absolute risk difference (per 1000 population over 10 years) of ischaemic heart disease and stroke in different diet groups in the EPIC-Oxford study

Outcome and diet groups*	Predicted incidence per 1000 population over 10 years†	Absolute risk difference per 1000 population over 10 years‡
**Ischaemic heart disease**		
Meat eaters	46.2 (43.8 to 48.7)	Reference
Fish eaters	40.4 (36.2 to 45.2)	−5.8 (−10.0 to −1.0)
Vegetarians	36.2 (33.1 to 39.5)	−10.0 (−13.1 to −6.7)
**Total stroke**		
Meat eaters	15.4 (14.1 to 16.8)	Reference
Fish eaters	17.5 (14.8 to 20.6)	2.1 (−0.6 to 5.3)
Vegetarians	18.3 (16.2 to 20.8)	3.0 (0.8 to 5.4)

* Meat eaters were participants who reported eating meat, regardless of whether they ate fish, dairy, or eggs; fish eaters were participants who did not eat meat but did eat fish; and vegetarians included vegans.

† For meat eaters, calculated as (1−S_r_)×1000, where S_r_=(1−observed incidence in meat eaters)^10^, and represents the predicted 10 year survival rate in the meat eaters. For all other diet groups, calculated as (1−S_r_^HR^)×1000, where HR represents the hazard ratio or confidence intervals of the hazard ratio for each outcome in that diet group, and S_r_^HR^ represents the predicted 10 year survival (that is, non-incidence) rate in the diet group.

‡ Calculated as the difference between the predicted incidence per 1000 population over 10 years between each diet group and the meat eaters.

### Sensitivity and subgroup analyses

In sensitivity analyses using baseline diet group only, excluding participants with less than five years of follow-up, including participants recruited via the postal method only, and performing multiple imputations for missing covariates, the results were similar (supplementary table 5). When the analyses were censored at age 70 or limited to those above age 70 (setting entry time at age 70) respectively, a significantly higher rate of haemorrhagic stroke was observed in vegetarians who had an event above the age of 70, but not in those who had an event before age 70 or were censored at that age. In subgroup analyses, significantly lower rates of ischaemic heart disease were observed among fish eaters only in those aged less than 60 years at recruitment, but lower rates were observed in vegetarians in both age groups (supplementary table 6). No other significant interactions by subgroups were observed for the association between diet group and ischaemic heart disease or stroke (supplementary tables 6-7).

## Discussion

The present study examines the risks of both ischaemic heart disease and stroke, including subtypes, in meat eaters, fish eaters, vegetarians, and vegans in a cohort with a large proportion of non-meat eaters. We observed lower rates of ischaemic heart disease in fish eaters and vegetarians than in meat eaters, which appears to be at least partly due to lower body mass index and lower rates of high blood pressure, high blood cholesterol, diabetes associated with these diets. Conversely, vegetarians had higher risks of haemorrhagic and total stroke.

### Comparison with other studies

In a collaborative meta-analysis of five previous prospective studies (Adventist Mortality Study, Health Food Shoppers Study, Adventist Health Study, Heidelberg Study, Oxford Vegetarian Study) with median recruitment between 1960 and 1980, vegetarians had a 24% lower rate ratio of death from ischaemic heart disease (0.76, 95% confidence interval 0.62 to 0.94) than non-vegetarians.^5^ In other analyses of death from ischaemic heart disease, the death rate ratios comparing vegetarians with non-vegetarians were 0.99 (0.79 to 1.23) in a combined analysis of EPIC-Oxford and the Oxford Vegetarian Study,^6^ 0.70 (0.41 to 1.18) in the German Vegetarian Study,^27^ and 0.81 (0.64 to 1.02) in the Adventist Health Study 2.^7^ In a recent meta-analysis that pooled the estimates from these previous prospective studies, including a previous report from the EPIC-Oxford study that only compared vegetarians with non-vegetarians (meat and fish eaters combined),^8^ the risk ratio for ischaemic heart disease comparing vegetarians with non-vegetarians was 0.75 (0.68 to 0.82).^28^

For stroke, previous analyses, including one study that included EPIC-Oxford data but with fewer cases than the present study,^6^ reported no significant differences in stroke mortality by diet group,^5^ ^6^ and pooled analyses showed a similar result.^28^ However, previous studies only reported on stroke mortality,^5^ ^6^ which might be strongly influenced by treatment as well as the underlying disease risk,^28^ and no studies were found which reported on the two subtypes of stroke.

### Interpretation of results and implications

The results of the present study showed that fish eaters and vegetarians had lower risks of ischaemic heart disease than meat eaters. The associations were attenuated after adjustment for self reported high blood pressure, high blood cholesterol, diabetes, and body mass index, which suggests that part of the associations might be attributed to these factors. However, the lower risk in vegetarians and vegans remained marginally significant after adjustment for all of these factors. The reason for such differences is not certain, but could be partly attributed to lower concentrations of low density lipoprotein cholesterol (LDL-C; or non-HDL-C concentrations as a surrogate) associated with meat-free diets,^29^ ^30^ ^31^ differences that were not fully accounted for by adjusting for self reported high blood cholesterol. Previous meta-analyses of prospective studies showed that lower concentrations of non-HDL-C or LDL-C were associated with lower risks of ischaemic heart disease.^26^ ^32^ Furthermore, both randomised trials of statin treatments^33^ and mendelian randomisation studies^30^ have confirmed a causal association of LDL-C with ischaemic heart disease. Hence, fish eaters and vegetarians, who have lower non-HDL-C (table 1) or LDL-C than meat eaters,^19^ ^34^ could have lower risks of ischaemic heart disease. However, the possible beneficial roles of generally healthier diets that included high intakes of fruit and vegetables,^35^ legumes,^36^ or fibre^37^ cannot be excluded, despite little change in the hazard ratios after adjustment for these individual components.

For stroke outcomes, the combined results from two randomised statin trials reported 21% higher risks of haemorrhagic stroke per 1 mmol/L reduction in LDL-C,^29^ ^38^ which are consistent with results of observational studies of cholesterol concentrations and haemorrhagic stroke.^39^ ^40^ ^41^ A recent study that included observational and genetic evidence from China alongside the trial evidence from western countries has suggested this inverse association between LDL-C and haemorrhagic stroke might be causal.^42^ These previous studies corroborate the findings of the present study, indicating that vegetarians, who have relatively low LDL-C, had higher risks of haemorrhagic stroke. For ischaemic stroke, previous prospective studies and randomised trials have consistently shown weak positive associations of non-HDL-C or LDL-C levels with risk,^26^ ^32^ ^33^ ^38^ which is supported by recent mendelian randomisation studies.^31^ ^42^ This apparent discordance between previous evidence and those of the present study for LDL-C levels and ischaemic stroke suggests that other dietary factors associated with the lack of animal food consumption could contribute to the observed associations.

Results of several studies in Japan, showing that individuals with a very low intake of animal products had an increased incidence and mortality from haemorrhagic and total stroke, and also a possibly higher risk of ischaemic stroke mortality,^43^ ^44^ ^45^ ^46^ suggest that some factors associated with animal food consumption might be protective for stroke. Vegetarians and vegans in the EPIC-Oxford cohort have lower circulating levels of several nutrients (eg, vitamin B_12_,^47^ vitamin D,^48^ essential amino acids,^49^ and long chain n-3 polyunsaturated fatty acids^50^), and differences in some of these nutritional factors could contribute to the observed associations.^45^ ^51^ ^52^ ^53^ ^54^ Serum concentrations of these nutritional factors and non-HDL-C have only been measured in a subset of the EPIC-Oxford cohort, and therefore their role in the observed associations of vegetarian diets with ischaemic heart disease or stroke cannot be accurately determined in the current context, but should be further investigated.

High blood pressure is an established major risk factor for both ischaemic heart disease and stroke,^24^ ^55^ and recent evidence confirmed this is true for all age groups, especially for haemorrhagic stroke.^56^ However, given that the vegetarians in the EPIC-Oxford study had lower blood pressure than the meat eaters, and the fact that the association between diet group and stroke risk became slightly stronger after adjustment for history of high blood pressure, this factor is unlikely to account for the higher risks of haemorrhagic and total stroke observed among vegetarians in the present study. Furthermore, although differential treatment of cardiovascular risk factors including hypertension in the different diet groups could influence their subsequent disease risk, this does not appear to be the case in the EPIC-Oxford study; previous analyses in this cohort have shown that although vegetarians had lower use of drug treatment overall than non-vegetarians, no significant differences were seen in the use of specific treatments for high blood pressure, high blood cholesterol, or diabetes by diet group, among those individuals who reported a diagnosis of these conditions.^57^

Misclassification of stroke cases, such as the misclassification of haemorrhagic stroke as ischaemic stroke, cannot be excluded as a possible explanation for the possibly discrepant results for ischaemic stroke, although previous adjudication studies of stroke types confirmed the reliability of hospital admission records and death certificates in the UK for diagnosis of stroke types in the UK population over this calendar period.^58^ ^59^ Although age is also an important risk factor for both ischaemic heart disease and stroke, and meat eaters in the present study were on average 10 years older than the vegetarians, age is known accurately in our cohort, and therefore any potential confounding or cohort effect is accounted for by the analyses that use age as the underlying time variable and further adjust for calendar year of entry.

The current study focused on the examination of risks associated with predefined dietary groups, and therefore the relative contributions of individual foods have not been assessed. Future research could benefit from dose-response analyses of foods that distinguish the diet groups, including meat, fish, dairy, and eggs, to identify possible optimal levels of consumption for balancing risks from different outcomes. Such analyses might be better performed in other large scale cohorts with greater numbers of people who consume these foods, or pooled analyses from multiple cohorts with varying levels of consumption. Methods such as diet optimisation modelling or linear modelling^60^ ^61^ could also be explored to identify optimal diets for disease prevention while respecting individual dietary choices.

### Strengths and limitations

The strengths of this study include a large sample size, a long follow-up, and outcome ascertainment by linkage to medical records that minimised the loss to follow-up. Because the exposure of interest was distinct diet groups, defined by the exclusion of animal foods, the chance of misclassification of exposure was relatively low. Long term adherence to diet groups in the cohort was also generally high, and where possible we updated the exposure and important confounders at 14 year follow-up to allow for any changes. The analyses also included adjustment for multiple confounders, assessment of several possible mediators, and sensitivity analyses to confirm the robustness of the results. 

Among the limitations of the present study, diet group was self reported, and reasons for choosing each diet were not recorded. Changes in diet group or other behaviours not captured by the follow-up were also possible, and the composition of vegetarian diets could have changed during follow-up owing to increasing availability of vegetarian foods, but differences in nutrient intakes between the diet groups were similar at baseline and at follow-up.^9^ ^62^ Reverse causality is possible but not likely, because the results were similar after we excluded the first five years of follow-up, and most participants had followed their current diet (eg, vegetarian) for more than five years at the time of recruitment. Information on drug treatment use (including statins) at recruitment was not available. As with all observational studies, residual confounding from either dietary or non-dietary factors is possible, which might be particularly relevant if results were of borderline significance. Generalisability could be limited, because the present study was based predominantly on white European individuals.

### Conclusions

Overall, the present study has shown that UK adults who were fish eaters or vegetarians had lower risks of ischaemic heart disease than meat eaters, but that vegetarians had higher risks of stroke. Future work should include further measurements of circulating levels of cholesterol subfractions, vitamin B_12_, amino acids, and fatty acids in the cohort to identify which factors might mediate the observed associations. Additional studies in other large scale cohorts with a high proportion of non-meat eaters are needed to confirm the generalisability of these results and assess their relevance for clinical practice and public health.

**What is already known on this topic**

Vegetarian and vegan diets have become increasingly popular in recent years, but the potential benefits and hazards of these diets are not fully understood
Previous studies of two diet groups have reported that vegetarians have lower risks of ischaemic heart disease than non-vegetarians
However, no evidence has been reported of a difference in the risk of mortality from stroke, possibly because of limited available data and lack of available evidence on stroke subtypes

**What this study adds**

This study showed that fish eaters and vegetarians (including vegans) had lower risks of ischaemic heart disease than meat eaters
Vegetarians (including vegans) had higher risks of haemorrhagic and total stroke than meat eaters
Further research is needed to replicate these results in other populations and to identify mediators that might contribute to the observed associations

## Web extra

Extra material supplied by authors

**Web appendix:** Supplementary material

## Notes

Contributors: TYNT and TJK conceived and designed the research question. TYNT analysed the data and wrote the first draft of the manuscript; PNA created the figure and prepared the data for analysis; and PNA, KEB, AP-C, RCT, RC, and TJK provided input on data analysis and interpretation of results. All authors revised the manuscript critically for important intellectual content, and read and approved the final manuscript. TYNT is the guarantor. The corresponding author attests that all listed authors meet authorship criteria and that no others meeting the criteria have been omitted.

Ethical approval: The study protocol was approved by a multicentre research ethics committee (Scotland A research ethics committee), and all participants provided written informed consent.

Data sharing: The data access policy for the EPIC-Oxford study is available via the study website (www.epic-oxford.org/data-access-sharing-and-collaboration/).

The lead author (the manuscript’s guarantor) affirms that this manuscript is an honest, accurate, and transparent account of the study being reported; that no important aspects of the study have been omitted; and that any discrepancies from the study as planned (and, if relevant, registered) have been explained.
