# Supplementary material for: Risks of ischaemic heart disease and stroke in meat eaters, fish eaters, and vegetarians over 18 years of follow-up: results from the prospective EPIC-Oxford study
Source: BMJ. 2019 Sep 4;366:l4897. doi: 10.1136/bmj.l4897 (PMC6724406; doi:10.1136/bmj.l4897)
Supplement: Supplementary file 1 — Web appendix: Supplementary material [file tont046481.ww.pdf]

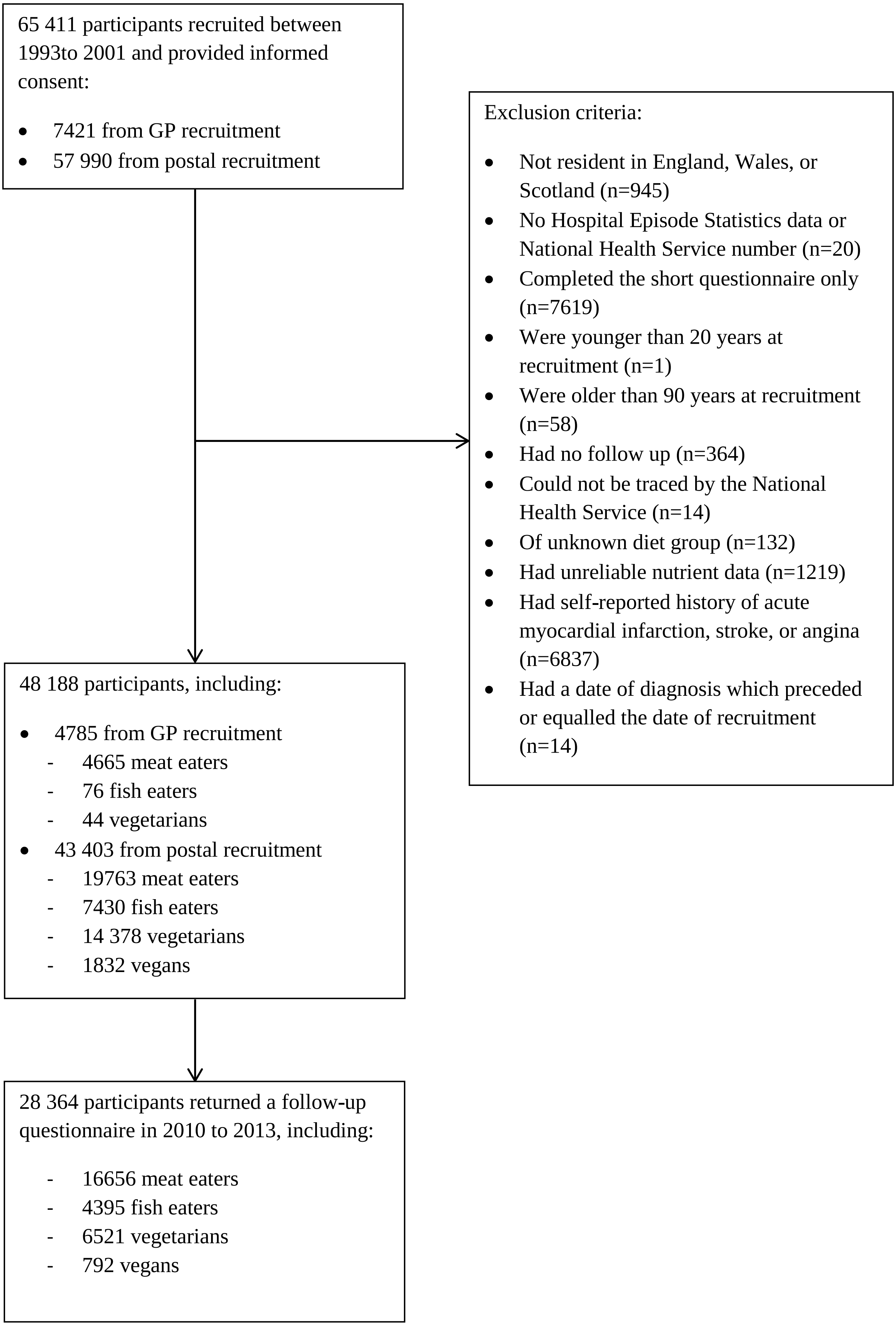

Supplementary figure 1: Participant flow chart of the study.

Supplementary table 1: Baseline characteristics of the overall cohort, and vegetarians and vegans in EPIC-Oxford.

**Characteristics**	**Overall cohort Max n=48 188**	**Diet groups**	
		**Vegetarians Max n=14 422**	**Vegans Max n=1832**
**Socio-demographic**			
Age, years (SD)	44.7 (13.8)	39.5 (13.1)	38.6 (13.2)
Sex, women (%)	36 899 (76.6)	11 063 (76.7)	1169 (63.8)
Top socio-economic quartile (%)[1]	10 408 (24.7)	2728 (21.6)	290 (18.0)
Degree education (%)	17 380 (38.5)	5949 (43.3)	749 (43.3)
**Lifestyle**			
Current smokers (%)	5404 (11.2)	1484 (10.3)	201 (11.0)
Alcohol consumption, g/day (SD)	9.8 (12.8)	9.4 (12.7)	8.5 (13.7)
Moderate/ high physical activity (%)	15 285 (35.6)	5088 (39.3)	761 (45.9)
Dietary supplement use, (%)[2]	26 958 (57.1)	8026 (56.6)	935 (52.1)
**Medical history, n (%)**			
Prior high blood pressure (% yes)	4422 (9.2)	850 (5.9)	85 (4.6)
Prior high blood cholesterol (% yes)	2216 (4.6)	317 (2.2)	28 (1.6)
Prior diabetes (% yes)	507 (1.1)	86 (0.6)	7 (0.4)
Receiving long term treatment for any illness (% yes)	11 721 (24.6)	2778 (19.4)	299 (16.4)
Oral contraceptive use (% yes)[3]	27 842 (75.8)	8791 (79.8)	829 (71.2)
Hormone replacement therapy use (% yes)[3]	6166 (16.9)	887 (8.1)	67 (5.9)
**Biological measurements, mean (95% CI)**[4]			
Body mass index (kg/m^2)	---	23.1 (23.1 to 23.2)	22.3 (22.1 to 22.5)
Systolic blood pressure (mmHg)	---	124.0 (123.5 to 124.6)	121.7 (120.5 to 123.0)
Diastolic blood pressure (mmHg)	---	76.1 (75.8 to 76.5)	74.4 (73.6 to 75.2)
Total cholesterol (mmol/L)	---	5.10 (5.03 to 5.17)	4.76 (4.67 to 4.85)
HDL cholesterol (mmol/L)	---	1.30 (1.27 to 1.32)	1.29 (1.25 to 1.32)
Non-HDL cholesterol (mmol/L)	---	3.80 (3.73 to 3.87)	3.47 (3.38 to 3.56)

Estimates shown are mean (SD), n (%), or adjusted means (95% confidence interval), as stated in left column.

1 Based on Townsend index.

2 Defined as regularly taking any vitamins, minerals, fish oils, fibre or other food supplements during the last 12 months.

3 In women only.

4 Body mass index was based on self-reported measures in the whole cohort. Blood lipids were measured in 674 vegetarians and 435 vegans, and blood pressure was measured in 3688 vegetarians and 676 vegans. Estimates were adjusted for the cross-stratification of gender and age at entry (5 year age groups), alcohol consumption (<1g, 1-7g, 8-15g, 16+ g/day), and physical activity (inactive, low activity, moderately active, very active, unknown).

Supplementary table 2: Food and nutrient intakes of vegetarians and vegans in EPIC-Oxford.

Foods or nutrients	Diet groups	
	**Vegetarians**	**Vegans**
	**Max n=14 422**	**Max n=1832**
**Foods**		
Dairy milk (ml/day)	260.6 (202.3)	---
Soya milk (ml/day)	32.7 (98.1)	228.4 (189.7)
Dairy cheese (g/day)	30.0 (25.1)	---
Total fresh fruit (g/day)	275.8 (221.0)	346.2 (345.1)
Total vegetables (g/day)	288.0 (157.8)	345.1 (193.4)
Legumes and soya foods (g/day)	70.6 (54.9)	104.7 (72.2)
Nuts and nut butter (g/day)	9.4 (13.9)	20.0 (26.5)
**Nutrients**		
Carbohydrates (% energy)	52.5 (6.6)	55.4 (7.8)
Protein (% energy)	13.6 (2.1)	13.3 (2.3)
Total fat (% energy)	30.5 (6.5)	28.1 (7.3)
Saturated fat (% energy)	10.7 (3.4)	6.5 (2.1)
Monounsaturated fat (% energy)	9.7 (2.5)	9.4 (3.4)
Polyunsaturated fat (% energy)	6.8 (2.3)	9.1 (2.9)
Dietary fibre (g/day)	21.6 (7.7)	25.9 (9.3)
Sodium (mg/day)	2673 (871)	2593 (986)
**Total energy (kJ/day)**	7871 (2213)	7359 (2346)

Estimates shown are mean (SD).

Supplementary table 3. Prospective associations between 4 diet groups and risk of cardiovascular diseases in EPIC-Oxford (n=48,188).

Outcome and diet groups	Cases	Person-years	Hazard ratios (95% confidence intervals)[1]	P-heterogeneity[2]
**Acute myocardial infarction**				
Meat eaters[3]	559	438,001	Reference	
Fish eaters[3]	84	132,168	1.00 (0.78 to 1.26)	
Vegetarians	129	246,889	0.91 (0.74 to 1.12)	
Vegans	16	31,911	0.77 (0.46 to 1.27)	0.63
**Ischaemic heart disease**				
Meat eaters[3]	2026	429,125	Reference	
Fish eaters[3]	298	130,816	0.87 (0.77 to 0.99)	
Vegetarians	429	245,309	0.77 (0.69 to 0.86)	
Vegans	67	31,629	0.82 (0.64 to 1.05)	<0.001
**Ischaemic stroke**				
Meat eaters[3]	340	438,418	Reference	
Fish eaters[3]	62	132,040	1.05 (0.80 to 1.39)	
Vegetarians	98	246,534	1.07 (0.84 to 1.36)	
Vegans	19	31,848	1.54 (0.95 to 2.48)	0.36
**Haemorrhagic stroke**				
Meat eaters[3]	173	438,418	Reference	
Fish eaters[3]	38	132,040	1.12 (0.78 to 1.61)	
Vegetarians	81	246,534	1.48 (1.11 to 1.97)	
Vegans	8	31,848	1.09 (0.53 to 2.26)	0.07
**Total stroke**				
Meat eaters[3]	678	438,418	Reference	
Fish eaters[3]	136	132,040	1.14 (0.94 to 1.38)	
Vegetarians	223	246,534	1.17 (1.00 to 1.38)	
Vegans	35	31,848	1.35 (0.95 to 1.92)	0.10

1 All analyses included age as the underlying time variable, and were stratified by sex, method of recruitment (general practice or postal), and region (7 categories), and adjusted for year of recruitment (per year), education (no qualifications, basic secondary [e.g. O level], higher secondary [e.g. A level], degree, unknown), Townsend deprivation index (quartiles, unknown), smoking (never, former, light, heavy, unknown), alcohol consumption(<1g, 1-7g, 8-15g, 16+ g/day), physical activity (inactive, low activity, moderately active, very active, unknown), dietary supplement use (no, yes, unknown), and oral contraceptive (no, yes, unknown) and hormone replacement therapy use (no, yes, unknown) in women.

2 P-heterogeneity represents significance of heterogeneity in risk between diet groups based on likelihood ratio tests.

3 Meat eaters were participants who reported eating meat, regardless of whether they ate fish, dairy, or eggs; fish eaters were participants who did not eat meat but did eat fish.

Supplementary table 4. Prospective associations between diet groups and risk of cardiovascular diseases in EPIC-Oxford, further adjusted for potential mediators of the associations, and possible relevant dietary factors (n =48 188).

Outcome and diet groups[1]	Hazard ratios (95% confidence intervals) with additional adjustment for[1]							
	Self-reported prior high blood pressure	Self-reported prior high blood cholesterol	Self-reported prior diabetes	Body mass index[4]	All potential mediators[5]	Fruit and vegetable intake[6]	Dietary fibre[6]	Total energy[6]
**Ischaemic heart disease**								
Meat eaters[2]	Reference	Reference	Reference	Reference	Reference	Reference	Reference	Reference
Fish eaters[2]	0.90 (0.79 to 1.02)	0.89 (0.78 to 1.00)	0.88 (0.78 to 1.00)	0.93 (0.82 to 1.05)	0.97 (0.85 to 1.10)	0.87 (0.77 to 0.99)	0.88 (0.78 to 1.00)	0.87 (0.77 to 0.99)
Vegetarians[2]	0.81 (0.73 to 0.90)	0.80 (0.72 to 0.89)	0.80 (0.72 to 0.89)	0.83 (0.75 to 0.92)	0.90 (0.81 to 1.00)	0.78 (0.70 to 0.87)	0.79 (0.71 to 0.88)	0.78 (0.70 to 0.86)
P-heterogeneity[3]	<0.001	<0.001	<0.001	0.003	0.16	<0.001	<0.001	<0.001
**Total stroke**								
Meat eaters[2]	Reference	Reference	Reference	Reference	Reference	Reference	Reference	Reference
Fish eaters[2]	1.16 (0.96 to 1.41)	1.13 (0.94 to 1.37)	1.14 (0.95 to 1.38)	1.15 (0.95 to 1.39)	1.17 (0.96 to 1.41)	1.13 (0.93 to 1.37)	1.14 (0.94 to 1.38)	1.14 (0.94 to 1.38)
Vegetarians[2]	1.24 (1.06 to 1.45)	1.19 (1.02 to 1.39)	1.21 (1.03 to 1.41)	1.21 (1.03 to 1.42)	1.25 (1.06 to 1.47)	1.18 (1.01 to 1.38)	1.19 (1.02 to 1.40)	1.20 (1.02 to 1.40)
P-heterogeneity[3]	0.02	0.07	0.04	0.04	0.02	0.09	0.08	0.06

1 Total analyses time was 836 979 person-years for IHD and 848 840 person-years for total stroke. All analyses included age as the underlying time variable, and were stratified by sex, method of recruitment (general practice or postal), and region (7 categories), and adjusted for year of recruitment (per year), education (no qualifications, basic secondary [e.g. O level], higher secondary [e.g. A level], degree, unknown), Townsend deprivation index (quartiles, unknown), smoking (never, former, light, heavy, unknown), alcohol consumption(<1g, 1-7g, 8-15g, 16+ g/day), physical activity (inactive, low activity, moderately active, very active, unknown), dietary supplement use (no, yes, unknown), and oral contraceptive and hormone replacement therapy use in women.

2 Meat eaters were participants who reported eating meat, regardless of whether they ate fish, dairy, or eggs; fish eaters were participants who did not eat meat but did eat fish; and vegetarians included vegans.

3 P-heterogeneity represents significance of heterogeneity in risk between diet groups based on Wald tests.

4 Body mass index was adjusted for categorically (<20, 20-22.5, 22.5-25, 25-27.5, 27.5+ kg/m^2, unknown).

5 All potential mediators included self-reported prior high blood cholesterol, self-reported prior high blood pressure, self-reported prior diabetes, and body mass index.

6 Fruit and vegetable intake, total fibre, and total energy were adjusted for continuously.

Supplementary table 5: Sensitivity analyses on prospective association between diet group and risk of ischaemic heart diseases and stroke.

	Hazard ratios (95% confidence intervals)[1]					
**Outcome and diet groups**	**Using baseline dietary and covariate data only N=48 188**	**Excluding participants with less than 5 years of follow-up N=46 834**	**Postal recruitment only N=43 403**	**Censoring at age 70 N=45 894**	**Setting entry time at age 70 N=13 975**	**Multiple imputation for covariates (10 datasets) N=48 188**
**Acute myocardial infarction**						
Meat eaters[2]	Reference	Reference	Reference	Reference	Reference	Reference
Fish eaters[2]	1.01 (0.79 to 1.28)	0.99 (0.76 to 1.28)	0.97 (0.76 to 1.24)	1.07 (0.76 to 1.51)	0.93 (0.67 to 1.30)	0.98 (0.77 to 1.25)
Vegetarians[2]	0.91 (0.75 to 1.11)	0.90 (0.73 to 1.12)	0.89 (0.73 to 1.09)	0.81 (0.60 to 1.09)	0.91 (0.69 to 1.19)	0.89 (0.73 to 1.09)
P-heterogeneity[3]	0.61	0.65	0.54	0.27	0.75	0.51
**Ischaemic heart disease**						
Meat eaters[2]	Reference	Reference	Reference	Reference	Reference	Reference
Fish eaters[2]	0.90 (0.79 to 1.02)	0.85 (0.74 to 0.98)	0.85 (0.75 to 0.97)	0.81 (0.68 to 0.98)	0.93 (0.78 to 1.10)	0.87 (0.77 to 0.99)
Vegetarians[2]	0.79 (0.71 to 0.88)	0.80 (0.72 to 0.90)	0.77 (0.69 to 0.86)	0.75 (0.65 to 0.87)	0.75 (0.65 to 0.87)	0.78 (0.70 to 0.87)
P-heterogeneity[3]	<0.001	<0.001	<0.001	<0.001	<0.001	<0.001
**Ischaemic stroke**						
Meat eaters[2]	Reference	Reference	Reference	Reference	Reference	Reference
Fish eaters[2]	1.06 (0.80 to 1.40)	1.05 (0.79 to 1.40)	1.05 (0.79 to 1.39)	1.35 (0.86 to 2.14)	0.94 (0.66 to 1.34)	1.06 (0.80 to 1.40)
Vegetarians[2]	1.16 (0.93 to 1.45)	1.08 (0.85 to 1.37)	1.12 (0.89 to 1.41)	1.46 (1.00 to 2.14)	0.93 (0.69 to 1.24)	1.13 (0.90 to 1.41)
P-heterogeneity[3]	0.44	0.80	0.61	0.12	0.85	0.58
**Haemorrhagic stroke**						
Meat eaters[2]	Reference	Reference	Reference	Reference	Reference	Reference
Fish eaters[2]	1.18 (0.82 to 1.68)	1.03 (0.68 to 1.55)	1.12 (0.78 to 1.62)	1.08 (0.67 to 1.73)	1.10 (0.61 to 1.96)	1.11 (0.77 to 1.60)
Vegetarians[2]	1.33 (1.00 to 1.77)	1.52 (1.12 to 2.06)	1.46 (1.10 to 1.94)	1.16 (0.79 to 1.69)	1.75 (1.15 to 2.67)	1.43 (1.08 to 1.89)
P-heterogeneity[3]	0.14	0.02	0.03	0.75	0.03	0.046
**Total stroke**						
Meat eaters[2]	Reference	Reference	Reference	Reference	Reference	Reference
Fish eaters[2]	1.19 (0.99 to 1.44)	1.06 (0.86 to 1.31)	1.13 (0.93 to 1.37)	1.30 (0.97 to 1.76)	1.04 (0.81 to 1.33)	1.14 (0.94 to 1.38)
Vegetarians[2]	1.19 (1.02 to 1.39)	1.19 (1.00 to 1.41)	1.20 (1.02 to 1.40)	1.27 (0.99 to 1.64)	1.07 (0.88 to 1.31)	1.20 (1.02 to 1.40)
P-heterogeneity[3]	0.04	0.14	0.06	0.09	0.78	0.06

1 All analyses included age as the underlying time variable, and were stratified by sex, method of recruitment (general practice or postal), and region (7 categories), and adjusted for year of recruitment (per year), education (no qualifications, basic secondary [e.g. O level], higher secondary [e.g. A level], degree, unknown), Townsend deprivation index (quartiles, unknown), smoking (never, former, light, heavy, unknown), alcohol consumption(<1g, 1-7g, 8-15g, 16+ g/day), physical activity (inactive, low activity, moderately active, very active, unknown), dietary supplement use (no, yes, unknown), and oral contraceptive and hormone replacement therapy use in women. Total N reported for each sensitivity analysis was based on N in the analyses for ischaemic heart disease if numbers varied.

2 Meat eaters were participants who reported eating meat, regardless of whether they ate fish, dairy, or eggs; fish eaters were participants who did not eat meat but did eat fish; and vegetarians included vegans.

3 P-heterogeneity represents significance of heterogeneity in risk between diet groups based on Wald tests.

Supplementary table 6: Risk of ischaemic heart disease by subgroups in different diet groups in EPIC-Oxford.

	Hazard ratios (95% confidence intervals)[1] Subgroups of variable			Test of difference between subgroups[6]
**Sex**	**Men**	**Women**		
N cases	1176	1644		
Meat eaters[2]	Reference	Reference		
Fish eaters[2]	0.78 (0.62 to 0.98)	0.92 (0.79 to 1.07)		χ^2=2.71
Vegetarians[2]	0.77 (0.66 to 0.91)	0.78 (0.68 to 0.90)		p=0.26
P-heterogeneity[3]	P=0.002	p=0.003		
**Age at recruitment**	**<60 years**	**≥60 years**		
N cases	1367	1453		
Meat eaters[2]	Reference	Reference		
Fish eaters[2]	0.75 (0.62 to 0.90)	1.03 (0.86 to 1.22)		χ^2=7.47
Vegetarians[2]	0.73 (0.63 to 0.85)	0.83 (0.71 to 0.97)		p=0.02
P-heterogeneity[3]	p<0.001	p=0.04		
**Body mass index**	**<25 kg/m^2**	**≥25 kg/m^2**		
N cases	1454	1262		
Meat eaters[2]	Reference	Reference		
Fish eaters[2]	0.95 (0.81 to 1.12)	0.93 (0.76 to 1.14)		χ^2=1.83
Vegetarians[2]	0.91 (0.79 to 1.04)	0.72 (0.60 to 0.87)		p=0.40
P-heterogeneity[3]	p=0.35	p=0.002		
**Smoking status**	**Never**	**Former**	**Current**	
N cases	1407	1062	335	
Meat eaters[2]	Reference	Reference	Reference	
Fish eaters[2]	0.87 (0.73 to 1.04)	0.90 (0.73 to 1.10)	0.79 (0.53 to 1.19)	χ^2=0.36
Vegetarians[2]	0.78 (0.67 to 0.90)	0.79 (0.67 to 0.94)	0.67 (0.47 to 0.97)	p=0.99
P-heterogeneity[3]	p=0.002	p=0.03	p=0.04	
**Presence of risk factors[4]**	**No**	**Yes**		
N cases	1833	965		
Meat eaters[2]	Reference	Reference		
Fish eaters[2]	0.88 (0.75 to 1.02)	1.02 (0.81 to 1.27)		χ^2=4.27
Vegetarians[2]	0.84 (0.74 to 0.95)	0.81 (0.66 to 0.99)		P=0.12
P-heterogeneity[3]	p=0.01	p=0.12		
**Receiving any treatment[5]**	**No**	**Yes**		
N cases	1558	1223		
Meat eaters[2]	Reference	Reference		
Fish eaters[2]	0.86 (0.73 to 1.02)	0.94 (0.77 to 1.15)		
Vegetarians[2]	0.78 (0.68 to 0.89)	0.84 (0.70 to 0.99)		χ^2=1.07
P-heterogeneity[3]	P<0.001	p=0.13		P=0.59

1. All analyses included age as the underlying time variable, and were stratified by sex, method of recruitment (general practice or postal), and region (7 categories), and adjusted for year of recruitment (per year), education (no qualifications, basic secondary [e.g. O level], higher secondary [e.g. A level], degree, unknown), Townsend deprivation index (quartiles, unknown), smoking (never, former, light, heavy, unknown), alcohol consumption (<1g, 1-7g, 8-15g, 16+ g/day), physical activity (inactive, low activity, moderately active, very active, unknown), dietary supplement use (no, yes, unknown), and oral contraceptive and hormone replacement therapy use in women.
2. Meat eaters were participants who reported eating meat, regardless of whether they ate fish, dairy, or eggs; fish eaters were participants who did not eat meat but did eat fish; and vegetarians included vegans.
3. P-heterogeneity represents significance of heterogeneity in risk between diet groups based on Wald tests.
4. Presence of one or more of self-reported prior high blood pressure, high blood cholesterol, or diabetes.
5. Based on responses to the question: "Are you receiving long-term treatment for any illness or condition?"
6. Test of interaction by sex, age at recruitment, body mass index, smoking, and presence of risk factors were performed by adding appropriate interaction terms to the Cox models, and testing for statistical significance of interaction across strata using likelihood ratio tests.

Supplementary table 7: Risk of stroke by subgroups in different diet groups in EPIC-Oxford.

	**Hazard ratios (95% confidence intervals)[1]** Subgroups of variable			**Test of difference between subgroups[6]**
**Sex**	**Men**	**Women**		
N cases	304	768		
Meat eaters[2]	Reference	Reference		
Fish eaters[2]	1.10 (0.73 to 1.65)	1.15 (0.93 to 1.43)		χ^2=2.81
Vegetarians[2]	0.99 (0.73 to 1.34)	1.28 (1.07 to 1.53)		p=0.25
P-heterogeneity[3]	p=0.88	p=0.03		
**Age at recruitment**	**<60 years**	**≥60 years**		
N cases	405	667		
Meat eaters[2]	Reference	Reference		
Fish eaters[2]	1.13 (0.84 to 1.52)	1.14 (0.89 to 1.46)		χ^2=0.05
Vegetarians[2]	1.19 (0.93 to 1.52)	1.20 (0.98 to 1.47)		p=0.98
P-heterogeneity[3]	p=0.34	p=0.17		
**Body mass index**	**<25 kg/m^2**	**≥25 kg/m^2**		
N cases	659	369		
Meat eaters[2]	Reference	Reference		
Fish eaters[2]	1.17 (0.93 to 1.47)	1.20 (0.83 to 1.72)		χ^2=0.96
Vegetarians[2]	1.16 (0.96 to 1.41)	1.29 (0.96 to 1.73)		P=0.62
P-heterogeneity[3]	p=0.20	p=0.19		
**Smoking status**	**Never**	**Former**	**Current**	
N cases	597	377	90	
Meat eaters[2]	Reference	Reference	Reference	
Fish eaters[2]	1.19 (0.92 to 1.54)	1.01 (0.73 to 1.40)	1.33 (0.69 to 2.55)	χ^2=2.91
Vegetarians[2]	1.19 (0.96 to 1.47)	1.24 (0.96 to 1.61)	1.27 (0.71 to 2.28)	P=0.57
P-heterogeneity[3]	p=0.17	p=0.25	p=0.59	
**Presence of risk factors[4]**	**No**	**Yes**		
N cases	740	322		
Meat eaters[2]	Reference	Reference		
Fish eaters[2]	1.23 (0.98 to 1.53)	1.01 (0.68 to 1.49)		χ^2=2.59
Vegetarians[2]	1.20 (1.00 to 1.44)	1.44 (1.06 to 1.96)		P=0.27
P-heterogeneity[3]	p=0.07	p=0.06		
**Receiving any treatment[5]**	**No**	**Yes**		
N cases	600	445		
Meat eaters[2]	Reference	Reference		
Fish eaters[2]	1.22 (0.95 to 1.56)	1.13 (0.83 to 1.53)		
Vegetarians[2]	1.31 (1.07 to 1.60)	1.17 (0.90 to 1.52)		χ^2=0.60
P-heterogeneity[3]	p=0.03	p=0.45		P=0.74

1 All analyses included age as the underlying time variable, and were stratified by sex, method of recruitment (general practice or postal), and region (7 categories), and adjusted for year of recruitment (per year), education (no qualifications, basic secondary [e.g. O level], higher secondary [e.g. A level], degree, unknown), Townsend deprivation index (quartiles, unknown), smoking (never, former, light, heavy, unknown), alcohol consumption (<1g, 1-7g, 8-15g, 16+ g/day), physical activity (inactive, low activity, moderately active, very active, unknown), dietary supplement use (no, yes, unknown), and oral contraceptive and hormone replacement therapy use in women.

2 Meat eaters were participants who reported eating meat, regardless of whether they ate fish, dairy, or eggs; fish eaters were participants who did not eat meat but did eat fish; and vegetarians included vegans.

3 P-heterogeneity represents significance of heterogeneity in risk between diet groups based on Wald tests.

4 Presence of one or more of self-reported prior high blood pressure, high blood cholesterol, or diabetes.

5 Based on responses to the question: “Are you receiving long-term treatment for any illness or condition?”

6 Test of interaction by sex, age at recruitment, body mass index, smoking, and presence of risk factors were performed by adding appropriate interaction terms to the Cox models, and testing for statistical significance of interaction across strata using likelihood ratio tests.
